# Heat Shock Proteins Are Essential Components in Transformation and Tumor Progression: Cancer Cell Intrinsic Pathways and Beyond

**DOI:** 10.3390/ijms20184507

**Published:** 2019-09-11

**Authors:** Benjamin J. Lang, Martín Eduardo Guerrero-Giménez, Thomas L. Prince, Andrew Ackerman, Cristina Bonorino, Stuart K. Calderwood

**Affiliations:** 1 Department of Radiation Oncology, Beth Israel Deaconess Medical Center, Harvard Medical School, Boston, MA 02215, USA; bjlang@bidmc.harvard.edu; 2 Laboratory of Oncology, Institute of Medicine and Experimental Biology of Cuyo (IMBECU), National Scientific and Technical Research Council (CONICET), Mendoza 5500, Argentina; martiguerrerog89@gmail.com; 3 Department of Functional Genomics, Geisinger Medical Center, Danville, PA 17822, USA; tprince@geisinger.org (T.L.P.); aackerman@geisinger.org (A.A.); 4 Departamento de Ciências Básicas da Saúde, Universidade Federal de Ciências da Saúde de Porto Alegre, Porto Alegre 90010, Brazil; cbonorino@ucsd.edu; 5 Department of Surgery, School of Medicine, University of California, San Diego, La Jolla, CA 92093, USA

**Keywords:** heat shock proteins, cancer, chaperome, molecular chaperones, tumor signaling, extracellular HSPs, proteotoxic stress, stress proteins in cancer, Hsp70, Hsp90

## Abstract

Heat shock protein (HSP) synthesis is switched on in a remarkably wide range of tumor cells, in both experimental animal systems and in human cancer, in which these proteins accumulate in high levels. In each case, elevated HSP concentrations bode ill for the patient, and are associated with a poor outlook in terms of survival in most cancer types. The significance of elevated HSPs is underpinned by their essential roles in mediating tumor cell intrinsic traits such as unscheduled cell division, escape from programmed cell death and senescence, de novo angiogenesis, and increased invasion and metastasis. An increased HSP expression thus seems essential for tumorigenesis. Perhaps of equal significance is the pronounced interplay between cancer cells and the tumor milieu, with essential roles for intracellular HSPs in the properties of the stromal cells, and their roles in programming malignant cells and in the release of HSPs from cancer cells to influence the behavior of the adjacent tumor and infiltrating the normal cells. These findings of a triple role for elevated HSP expression in tumorigenesis strongly support the targeting of HSPs in cancer, especially given the role of such stress proteins in resistance to conventional therapies.

## 1. Introduction: Molecular Chaperones, Protein Folding, and the Health of the Proteome

**Figure 1:**
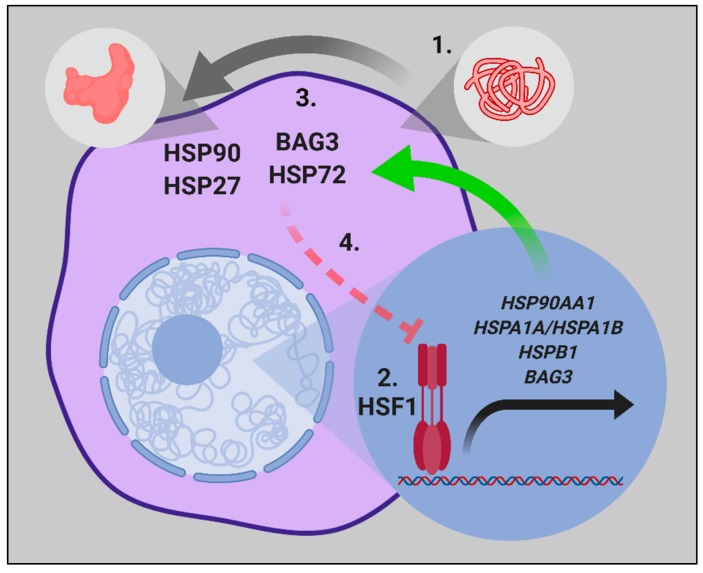
The central dogma of the heat shock proteins-1 (HSP1) mediated proteotoxic stress response. (**1**) Cellular stressors such as heat shock, oxidative stress, exposure to heavy metals, or proteasome inhibition, which induce increased levels of non-native protein conformations (proteotoxic stress) leads to the activation of HSF1. (**2**) HSF1 translocates to the nucleus, and HSF1 trimers rapidly bind to heat shock elements (HSE) in the promoter region of stress-inducible genes and transactivates mRNA expression. (**3**) Increased cytosolic HSP levels promote the refolding of proteins in non-native conformations to achieve the native functional protein structure. (**4**) As proteostasis is restored, a negative feedback loop exists, where HSP72 then inhibits the HSF1 activity further.

Heat shock proteins (HSP) are the products of a class of genes that become induced at an unprecedented amplitude during proteotoxic stresses, most notably, heat shock itself [1,2]. They belong to a handful of gene families that encode molecular chaperones, proteins that function to maintain the structural integrity of the proteome and thereby promote cell viability [2]. Molecular chaperone genes can be either synthesized constitutively or in the case of the inducible HSP genes, are either silent or weakly expressed in unstressed cells, but turn on at dramatic rates after stress, to cope with the crisis in the protein folding that ensues [3]. Inducible HSP genes each contain, within their promoters, binding sites for heat shock transcription factor 1 (HSF1), a protein that senses exposure to stress almost instantaneously, changes from a monomer to a trimer form, and powerfully induces HSP genes [4,5,6]. Here, we will discuss a subset of HSPs known to be significant in cancer, including Hsp27, Hsp70, and Hsp90, each regulated by HSF1 (Figure 1) [7]. Although roles for other chaperones and chaperonins are emerging, most studies have concentrated on these protein families, and thus our account is mostly focused on these three chaperones. It is still unclear to what degree the mechanisms of induction of HSP expression in cancer resemble the changes undergone during the heat shock response, and that question will be a point of discussion here.

### 1.1. HSF1 and the Heat Shock Response

Upon exposure of mammalian cells to heat shock, HSF1 monomers become activated, form trimers, and rapidly migrate to *HSP* gene promoters, leading to the transcription of the cohort of *HSP* genes (Figure 1) [6]. It is by far the most rapidly activated inducible transcriptional program in mammalian genomes, and HSF1 arrives upon the chromatin within 30 s of heat shock, as compared with the requirement of 10–15 min in rapidly-inducible “immediate early genes” such as c-fos, c-jun, and egr-1 after growth factor stimulation [8]. The trigger mechanisms involved in HSF1 activation by stress are still debated, although processes such as the direct sensing of heat shock by a HSF1 tertiary structure, the reversal of HSF1 repression exerted by HSPs in a feedback response, and multiple posttranslational modifications (PTMs) appear to play active roles [9,10,11,12,13,14]. The profile of the heat shock response program is similar in most cells, with the rapid activation of *HSP* transcription, stabilized high level expression of *HSP* mRNAs, and the persistent expression of HSPs, which may last for up to 100 h as the proteotoxic stress is resolved [2,3,15].

While the mechanisms of stress-induced HSF1 activation are yet to be fully defined, the activation of HSF1 in cancer is even less well understood in many malignant tissues, and nuclear HSF1 is observed in the absence of any external stress, a phenomenon that may account for the constitutive HSP expression observed in many cancer cells [16,17]. HSF1 activation has been shown to occur downstream of the growth factor stimulation and was, for instance, induced in mammary cancer by the cytokine heregulin, which activates Her2 signaling [18]. As the heat shock response is characteristically triggered by proteotoxic stresses, there has been much speculation that similar mechanisms may underlie *HSP* transcription in cancer cells [19,20]. Cancer cells often express mutated oncogenes that require a high-level chaperone expression to maintain stability and function, have undergone polyploidy, and have enhanced rates of protein synthesis, and thus may be under a net “folding pressure” [20]. While this would be a difficult hypothesis to test, Sherman and co-workers have indicated that protein unfolding is not increased in cancer [21]. At the moment, however, this “addicted to chaperones” theory is perhaps the most acceptable hypothesis available—implying that the heat shock response gradually increases in activity in many cancer cells, so as to counter the demand for folding exacted by the processes of transformation and tumor progression. HSF1 and HSPs, thus induced, may function mostly in oncogenic protein folding cascades, or may be free to take on new roles in tumorigenesis [21,22]. Indeed, the chromatin precipitation coupled with next generation sequencing (CHIP-Seq) analysis of HSF1 binding to chromatin in human cancer cells has revealed multiple transcriptional targets over and above the classical *HSP* genes themselves [23].

### 1.2. Mechanisms of Protein Folding and Chaperone Performance

**Figure 2:**
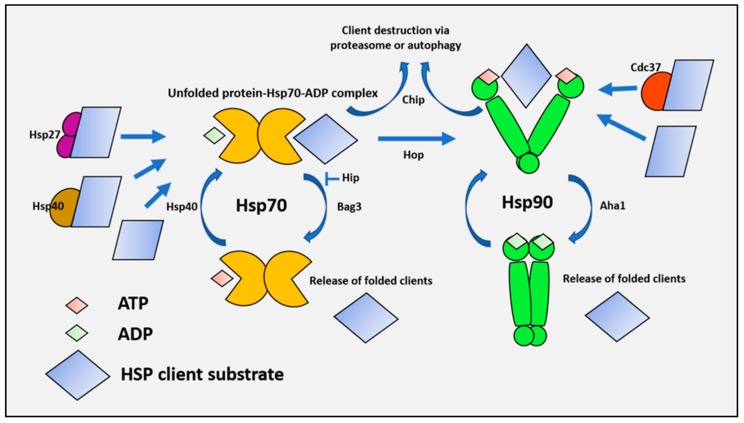
Integrated Hsp27, Hsp70, and Hsp90 functions promote proteostasis. There are multiple entry points into the cytosolic chaperone network, where substrates can be recruited by Hsp27, Hsp70 co chaperones (e.g., Hsp40 family members), and Hsp90 co chaperones (e.g., Cdc37), or are bound directly by Hsp70 or Hsp90. Unfolded client proteins are relayed from Hsp27 or Hsp40 to the ATP-bound Hsp70 complex under the direction of J domain co-chaperone DNAJ. Binding of the unfolded client triggers the innate ATPase activity of Hsp70 through allosteric interactions, leading to a high affinity complex containing Hsp70, client, and ADP. The complex then undergoes nucleotide exchange facilitated by binding the co-chaperone Bag3. ADP is exchanged for ATP, which lowers the affinity of Hsp70 for the bound client that is released. Alternative destinations for Hsp70 substrates include proteasomal degradation via *STUB1*/CHIP, or transfer via HOP for further folding by the Hsp90 complex.

Upon synthesis on the ribosome, proteins must fold correctly to their functional conformations within the crowded intracellular environment, which is no mean accomplishment [24]. Likewise, the proteins that become unfolded or aggregated particularly after stress are required to be refolded to maintain viability [1]. The functional conformation will be the lowest free energy state of the polypeptide, and thus folding to this shape is preferred [25]. However as non-native metastable protein conformations may also be assumed prior to achieving the native state, HSPs offer the possibility of escaping from these dead-end conformations, by lowering the activation energy required to reach the goal of functionality [26]. The HSPs each play subtly different roles in the complex process of folding the proteome. Hsp27, 70, and 90, to be discussed here for their participation in tumorigenesis, appear to operate in relay to deliver an optimally folded product [7,27]. HSPs in general appear to be able to recognize the general structural characteristics that are clues pointing to malfolding; for instance, hydrophobic residues are rarely found on the 3D surface of functional proteins, and when inappropriately exposed, can trigger protein aggregation with other hydrophobic residues. HSPs recognize and associate with hydrophobic regions of substrates, prevent their aggregation, and specific HSP species can also catalyze refolding or degradation [28]. Small HSPs such as Hsp27 are efficient in binding to unfolded proteins, and act to protect against the formation of larger insoluble aggregates through stable “holdase”-type associations, and although they are generally considered to lack an autonomous re-folding ability, they may indirectly catalyze refolding by directing substrates to larger Hsp70 complexes [29]. These properties seem to involve the ability of Hsp27 to transition between shorter and larger oligomers, a regulatory feature that is responsive to temperature and Hsp27 phosphorylation [30,31,32]. While transient Hsp27 interactions with non-native substrate conformations have been shown to protect against protein aggregation, the dissociation of Hsp27 from a more stable engagement with non-native intermediates is an inefficient process, and a dependence exists on other chaperones, including Hsp70, to facilitate Hsp27 release and substrate refolding [29,30,33,34]. The ability of Hsp27 to protect against protein aggregation in an ATP-independent manner may have particular importance in contexts such as heat shock, where the metabolic production of ATP is perturbed [35], and the functional capacity of ATP-dependent chaperones may be restricted or insufficient. A model has been proposed for contexts where the cellular need for refolding exceeds the capacity of the ATP-dependent chaperones, whereby Hsp27 harbors non-native intermediates in a “folding-permissive” state, which protects against the formation of insoluble protein aggregates, until they are progressively processed by Hsp70 and Hsp90 throughout the recovery phase [34]. Hsp70 can bind unfolded or partially folded polypeptides either associated with Hsp27, undergoing synthesis on ribosomes, or free in solution. Hsp70 cycles between ATP-bound, polypeptide-free, and ADP-polypeptide-bound states, in which substrate conformation can be re-organized to allow for the native conformation to be adopted (Figure 2).

A number of accessory proteins or co-chaperones catalyze various aspects of the Hsp70 folding cycle, including the detection of potential clients (J domain proteins such as Hsp40) and nucleotide exchange (Bag1, Bag3, and Hspbp1) [36]. These co-chaperone proteins, which increase the rates of the reactions that drive the chaperone cycle, may also participate in transformation and tumorigenesis (see review [36]; Figure 2).

Bag3 may be of particular significance both as a co-chaperone for Hsp70, and as an Hsp70-dependent component of the multiple cell signaling pathways important in tumorigenesis [21,37,38]. Bag-3 can bind both Hsp27 and Hsp70, and it is tempting to speculate a go-between role in the switch of clients from Hsp27 to Hsp70 [39]. There are several possible fates for a Hsp70 client, including release in its native form, degradation by the autophagy or proteasome pathways, or transfer to the TRiC/CCT or Hsp90 complexes for further folding (Figure 2) [19,40,41]. The transfer of the Hsp70 client substrates to Hsp90 is mediated by the co-chaperone HOP, which can bind to the C-terminal TPR binding domains found in both Hsp70 and Hsp90, and couple the two proteins, permitting the formation of a transient Hsp70-client protein-Hsp90-co-chaperone complex [19]. Hsp70 then dissociates from this complex, leaving Hsp90 and its co-chaperones to carry out the folding of the client to its native, functional form. The co-chaperone Cdc37 plays a key role in this process, and is required for the chaperoning of oncogenic kinases [42,43,44]. Then, after folding to its functional form, the client dissociates from Hsp90 to perform its functions in the cell, which may be appropriated in the processes of malignant transformation [45,46]. Recent studies carried out in vitro have shown a novel mechanism for the combinatorial functions of Hsp70 and Hsp90 [47,48]. Hsp70 is a powerful chaperone that binds rapidly to the multiple hydrophobic sequences that are exposed on the surfaces of unfolded proteins, preventing aggregation. However, this poses a problem for the ultimate folding of polypeptides, whose stability requires these hydrophobic residues to be packed in the interior. At physiological Hsp70 concentrations, these residues would be mostly sequestered by the chaperone, causing a block to the ultimate folding step. These investigators showed that the problem could be solved by the addition of Hsp90, which could take over the Hsp70 bound polypeptides, and allow folding to resume and hydrophobic residues to concentrate in the interior [47,48].

An alternative folding destination from the Hsp70 complex is the transfer to the TRiC/CCT chaperonin complex. CCT subunits form multiprotein folding complexes in the cytoplasm, and may offer an alternative pathway for the completion of folding [41,49]. The degree to which co-chaperones play a key role in directing HSP substrates to their destination is increasingly apparent, although the criteria that direct substrates to their respective destinations (i.e., trafficking from Hsp70 to the Hsp90 complex as opposed to the CCT complex) remains largely unknown. Nevertheless, as transformed cells were found to have a dependence on elements of the HSF1/HSP heat shock response pathway, it has become increasingly apparent that many tumorigenic processes are acutely dependent on the combinatorial mechanisms of folding that exist between molecular chaperones and co-chaperones. In particular, Hsp27, Hsp70, and Hsp90 have been heavily studied, and their importance for tumorigenesis is well established; the mechanistic basis of this relationship, however, continues to be misunderstood.

### 1.3. Chaperones Work in a Coordinated Manner to Brace Cancer Cell Survival—The Chaperome in Cancer

**Figure 3:**
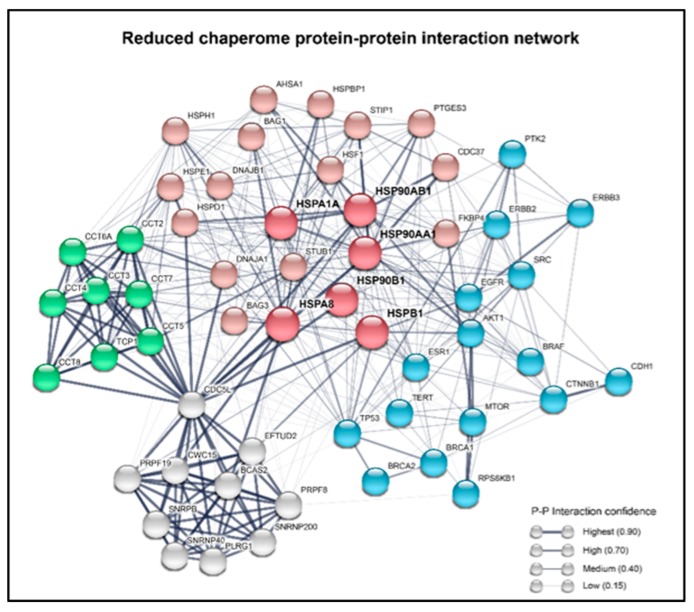
A reduced chaperome network is shown with the representation of the physical connections between the chaperome and the various proteins that can powerfully influence tumorigenesis. A summary of the protein–protein interaction network of the major HSPs. In the network, the nodes represent the most important constituents of the chaperome, which are connected with other proteins by edges of varying width. Red nodes are code for the main HSPs and co-chaperones, blue are well known cancer-related genes, the TRiC complex genes are shown in green, and the Prp19/CDC5L complex genes are in grey. The line thickness of the edges indicates the strength of the experimental data supporting a protein–protein interaction. The network was built using the STRING database (https://string-db.org) from the Swiss Institute of Bioinformatics and the European Molecular Biology Laboratory (EMBL).

Hsp70 and Hsp90 are key components within a larger “chaperome” network that comprises an abundant and evolutionary conserved family of proteins, including chaperones, co-chaperones, scaffolding proteins, folding enzymes, isomerases, and adaptor proteins, which systematically maintain optimal protein quality control [50,51,52]. A recent revelation has been the extent to which the chaperome network becomes functionally and physically integrated in contexts of proteostatic disruption or oncogenic transformation [53]. Under such stressful states, the chaperome becomes rearranged into a network of tightly interconnected HSPs and co-chaperones, forming stable high-molecular-weight complexes. At the heart of this network are HSP90 and HSC70, which act as nucleating sites for a series of co-chaperones that become physically incorporated into complexes with the larger Hsp90 and Hsp70 members. The coordinated function, regulation, and activities of these complexes have become increasingly clear, and the network together can be described as an “epichaperome”—a protein network that bridges the chaperome to different cellular pathways, and is vital for tumor survival [53]. The epichaperome, defined by formation stable, large HSP complexes, was found to be present within a majority of representative tumor cell samples in vitro, ex vivo, and in vivo, irrespective of the tissue of origin or genetic background. The formation of the epichaperome was found to be induced upon the expression of the MYC oncogene, and was suppressed upon MYC knockdown, thereby demonstrating the functional responsiveness of the epichaperome system upon oncogenic transformation [53]. Indeed, multiple tumor promoting molecular events, including those orchestrated by mutant TP53, ESR1, BRAF, vSrc, Met, ERBB2, Telomerase, numerous other kinases, and other cancer-related genes have a functional dependence on individual or multiple components of the chaperome (Figure 3) [54,55,56]. The functional importance of the epichaperome was further demonstrated by evidence indicating that this protein network potently enhances cell survival [53,57]. Of note, it was shown that the epichaperome network could compensate for a degree of Hsp90 inhibition, although the near-complete inhibition of Hsp90 led to a collapse of the epichaperome and cell death, illustrating the potential for targeting the epichaperome as a therapeutic strategy [53,57]. By taking the expression levels of the epichaperome into consideration, Chiosis and colleagues were able to identify two different types of cancer cells that were named “Type 1” (enriched with HSP90 high-molecular-weight complexes) and “Type 2” cells (with low or no presence of the HSP90 complexes), which showed a different binding affinity and sensitivity to chaperone inhibitors, and thereby provided a proof of concept that the epichaperome can be targeted with a selective toxicity to the transformed cells that have an apparent higher dependence on the network [53].

A significant proportion of chaperones and co-chaperones share similar protein functions and a high degree of homology with other members of the respective families. This feature has been suggested to contribute to the capacity of the chaperome to functionally compensate for the impaired activity of individual members of the network [58]. Nevertheless, 55 of 332 chaperone genes were shown to be essential for viability in K562 human leukemia cells, which is in agreement with findings from Rodina et al., and supports the notion that key nodes within the chaperome network may be targeted in cancer in order to achieve toxicity to transformed cells [26,52,59]. This has previously been exemplified by studies demonstrating the increased toxicity of Hsp90 inhibitors upon the co-inhibition of Hsp70 [60,61].

In addition to the integrated functions between individual epichaperome members, the expression of the respective protein encoding genes appears to be regulated in a coordinated manner. The extent to which this occurs in human breast tumors was recently revealed by Zoppino et al., where distinct HSP expression patterns were identified across the TCGA and METABRIC gene expression datasets, and were strongly related with patient survival [62,63]. These different patterns allowed ofr the characterization of tumors into three different HSP subtypes, named HSP-Clust I, HSP-Clust II, and HSP-Clust III. Interestingly, HSP-Clust I was associated with a better patient survival outcome, while HSP-Clust II (enriched with Basal-like tumors) and HSP-Clust III showed higher mortality rates and were particularly enriched with the epichaperome genes *HSP90AA1*, *HSPH1,* and *HSPA8*. Of note, these genes were also clustered together with other HSP90 and HSP70 genes, such as *HSP90AB1*, *HSP90B1*, *HSPA14*, *HSPA4*, *HSPA5, HSPA9, HSPA1A,* and *HSPA1B,* and multiple genes of the chaperonin family including *HSPE1* and *HSPD1,* as well as all genes encoding the TRiC complex (*TCP1* and *CCT2-CCT8*). Together, these studies indicated that epichaperome genes are expressed, and their respective proteins function in coordinated ways to augment tumor survival.

The coordinated expression of HSPs was also demonstrated by Esfahani et al. in a study that systematically analyzed the proteostatic network of more than 10,000 tumor biopsies of 22 different solid tumors types, and found a consistent up-regulation of the HSP90, HSP70, Chaperonins, Prefoldins, and HSP100 gene families, describing two types of cancers according to the preferential up-regulation of the ATP-dependent chaperones [64].

A bioinformatic analysis of protein–protein interaction curated databases shows that indeed the mentioned genes physically interact with each other in a complex network of chaperones, co-chaperones, and client proteins (Figure 3). Altogether, these data suggest that cancers consistently display signatures of a coordinated active chaperome network, which confer tumor cells with an increased proteostatic capacity and survival benefits that go beyond the isolated function of each of the chaperones. This is evidence pointing to the potential additional therapeutic value and/or importance in targeting the epichaperome as a network that may exceed targeting the individual chaperome members as a therapeutic strategy in cancer cells with a high chaperome expression. Thus, although in the following pages we discuss the individual roles of HSPs in cancer properties, it is expected that future studies will place further emphasis on the combinatorial properties of the chaperome. 

## 2. HSPs and the Cell-Intrinsic Properties of Malignant Cells

### 2.1. The Acquired Properties of Malignant Cells

Malignant transformation involves profound changes in the cellular phenotype that occur in the change from a differentiated cell protected by multiple tumor suppressing mechanisms that prevent reversion, to the properties that lead to cancer [65]. In the case of carcinomas, the epithelial cells that form the basis of tissues need to acquire the unfamiliar properties of proliferation in the absence of growth stimuli; resistance to oncogene-induced apoptosis and senescence; de novo angiogenesis, mobility and invasion of surrounding tissues; and most sinister of all, the metastasis and colonization of distant organs [65]. These properties are prohibited for differentiated cells, and therefore, malignant transformation may require multiple changes in regulatory molecules, by mutation and over-expression, in order to break the normal phenotypic controls and attain the full malignant phenotype [66,67]. However, in addition to such mutations and changes in cell intrinsic regulation, there is increasing evidence for a major role for stromal cells in the tumor milieu in programming the epithelial cells towards the malignant phenotype and protecting them from attack by the immune system [68,69,70,71]. Our task here is to analyze the mechanisms by which HSPs facilitate the long multistep process of tumorigenesis.

### 2.2. HSPs Shore Up the Shared Pro-Malignant Properties of Malignant Cells

#### 2.2.1. Unrestricted Proliferation

Most normal cells are quiescent because of a lack of exposure to the growth factors essential for proliferation. Growth factors bind to receptors, usually tyrosine kinases, and lead to signaling cascades that permit waves of gene expression, permitting proliferation [72]. Escape from this restriction requires either an activating mutation of growth factor receptors or of downstream signaling components. Thus, the receptor genes for EGFR, PDGF, FGF, HER2, and HER3, and signaling proteins such as Ras, PI-3 kinase, and PTEN, often become modified by deregulation, to serve as oncogenes [65,72]. Many of these proteins have relatively unstable conformations, which are associated with a proficient catalytic activity, and are heavily dependent on chaperoning by Hsp90 [19,45,73]. Therefore, the increases in Hsp90 closely associated with malignancy can permit the operation of growth factor receptor signaling cascades and proliferation in the absence of external growth stimulus [19,46]. This requirement for the stabilization of growth promoting oncoproteins forms the basis for the development of Hsp90 inhibitors, which have proceeded to the stage of clinical trial [20,74]. Given the dependence of such key proliferative signals upon Hsp90, it can be thought of as the “godfather of tumor proliferation”, a relationship that is exemplified by the well-established potent restriction of cancer cell proliferation induced by Hsp90 inhibitors [75,76]. In addition, Hsp27 has been shown to play a key role in cell cycle progression, by repressing the cell cycle inhibitory proteins E2F-4 and p130, and permitting the synthesis of multiple cell cycle proteins (CCNA2, CCNB1, CCNB, CDC25C, CDC3A, and CDK1) [77]. The perturbation of Hsp70 activity also inhibits the stabilization of mitogenic signal transducers that include BRAF [78].

#### 2.2.2. Evasion of Programmed Cell Death

The expression of oncogenes in otherwise normal cells can precipitate programmed cell death (PCD) [79]. Therefore, one of the earliest changes, found in the majority of cancers, is the blockade of the pathways that govern PCD [65]. This process can occur through multiple mechanisms, including the increased expression of the PCD inhibitor BCL2, or the loss of function mutation of the p53 gene, a key mediator of PCD that occurs downstream of DNA damage [80,81]. HSPs are involved in these mechanisms, and Hsp70 can bind to and stabilize mutant p53 alleles, permitting them to inhibit the wild type p53 alleles, and mimicking the effects of the homozygous mutation and loss of function [82]. In addition, high levels of Hsp90, Hsp70, and Hsp27 can directly inhibit multiple steps in the pathways of PCD [83,84,85]. HSPs thus reinforce the effects of p53 mutation, as well as leading to the direct inhibition of PCD. Both Hsp70 and Hsp27 have been shown to bind directly to intermediates in the PCD pathways, inhibiting multiple steps [86,87,88]. The roles of HSPs in inhibiting PCD in cancer may thus reflect their activities in the heat shock response, inhibiting cell death so as to enable the repair of lethal protein damage. In addition, both Hsp70 and Bag3 can suppress PCD downstream of HSF1 in cancer [38]. The simultaneous activation of proliferation, as described in (a), combined with the inhibition of PCD, would be likely to increase the rate of tumor growth. However, as most cancer cells are denied access to the PCD pathways because of Bcl2 family protein increases and p53 changes, and usually die by default death pathways such as mitotic catastrophe and necrosis, the impact of HSPs at this level is unclear [89].

#### 2.2.3. Hurdling Replicative Senescence

Another early check to the emergence of cancer is replicative senescence, a process that occurs within fifty cell divisions in normal cells because of the loss of telomere length at each cell division, a mechanism that times out the lifetime of the cell and its progeny [90]. Tumor initiation would thus be brought to a halt before the emergence of macroscopic cancers. However, cancer cells can overcome this hurdle by the use of the enzyme telomerase to patch-repair the telomeres and thwart senescence. Elevated levels of HSPs have some considerable influence in this process, and Hsp90 is known to stabilize the structure of telomerase in some cancers [91]. In addition, Hsp70 and Hsp27 can block the downstream pathways leading from telomere shortening to senescence by mechanisms including the inhibition of the ability of wild type p53 to initiate the senescence pathway [92,93]. Hsp72 inhibited both the p53-dependent pathway triggered by the expression of oncogenic PI3K and the p53-independent senescence pathway induced by the expression of the oncogene Ras [94]. Indeed, In the HER2/*neu* model of spontaneous mouse mammary carcinogenesis, the inactivation of Hsp72 by homologous recombination prevented the formation of tumors in 100% of mice because of onset of senescence [95].

#### 2.2.4. Angiogenesis and Glucose Metabolism

Mammalian cells require access to the circulation in order to acquire essential oxygen and nutrients. Once tumors grow and begin to invade into their tissues of origin, they become remote from the microcirculation. Indeed, pronounced hypoxia is observed in many macroscopic tumors [96]. However, most tumors are able to assemble a microcirculation of sorts by de novo angiogenesis, through the sensing of hypoxia by the hypoxia inducible factor (HIF) family transcription factors, and the synthesis and release of angiogenic cytokines, such as VEGF, that stimulate vascular endothelial cell proliferation [97,98]. HSF1 and HSPs Hsp27, Hsp70, and Hsp90 may contribute to angiogenesis though increases in the HIF1 expression, the chaperoning of VEGF receptors, and increasing the potency of VEGF signaling [21,99,100,101]. In addition to hypoxia, tumor cells also undergo a metabolic transformation in the metabolism, converting from preferred use of oxidative phosphorylation to aerobic glycolysis, a switch that evidently confers a growth advantage [102]. Indeed, elevated levels of both Hsp90 and Hsp70 have been shown to reduce oxidative phosphorylation and increase aerobic glycolysis in tumor cells [76,103].

#### 2.2.5. Invasion and Metastasis

Epithelial cells are tightly tethered in their appropriate location through a number of adhesion and junctional molecules in order to form stable, highly organized tissues [104]. At some stage in transformation however, the transformed cells become mobile, able to migrate through the tissue of origin and invade the surrounding structures [105]. These properties appear to involve a change in phenotype, known as the epithelial to mesenchymal transition (EMT), in which an epithelial cell can lose its characteristically cuboid, columnar morphology, and can adopt a more spindle-like appearance, common to cells of a mesenchymal origin [106]. Underlying EMT is a switch in expression from epithelial marker genes such as E-Cadhedrin, claudin, and surface beta-catenin to mesenchymal markers, most typically vimentin, indicators of a transcriptional switch orchestrated by a series of transcription factors that can include SNAIL, SLUG, TWIST, or ZEB1, depending on the cell type and context [105]. The activation of an EMT program can confer tumor cells with relatively motile, PCD-resistant properties that ultimately increase the potential for them to progress through the organ of origin, and ultimately replace the differentiated normal tissues. This process is typically accompanied by the secretion from tumor stromal cells, such as the cancer associated fibroblasts (CAFs) of growth factors and extracellular matrix (ECM) proteins that include fibrillar collagens, which form “metastasis highways” in the tissue. The production of ECM remodeling enzymes such as MMP2 by the tumor cells themselves, or CAFs, then permit the passage of cells through the ECM and out of the home tissue [107,108]. Some of the invading, mobile tumor cells are also able to escape the tissue of origin into lymph ducts and the microcirculation, survive passage through these vessels, gain entry into distant organs, and grow as de novo tumors among the colonized cells [109]. Hsp70 has been shown to be essential for invasion and metastasis in the PyMT-driven mouse spontaneous MMT mammary tumor and in human mammary lines, suggesting a fundamental role for the chaperone in this process [22,110]. These events seem to involve the expression of the c-MET oncogene, as well as its ligand HGF, and phospho-MET, the activated form of c-MET, was absent from Hsp72-deficient MMT tumors. Likewise, elevated Hsp27 was required for the invasion and metastasis triggered by HGF, and presumably downstream MET activation [111,112], as well as other mechanisms [113]. c-MET is a potent oncogene in many cell types, and its dependence on HSP expression for activation hints at one of the mechanisms underlying the roles of HSPs in cancer [114]. Hsp27 was also shown to be involved in the processing of matrix metalloproteinase 9 (MMP9), a key mediator of invasion [115]. Hsp27 seems of key importance at a number of steps in the metastatic cascade, including EMT, metastasis, and circulating tumor cells in prostate cancer, overall suggesting a versatile role for the chaperone in the secondary spread of cancer [116]. The perturbation of Hsp90 or Hsp70 disrupts key proteins for migration and invasion, including FAK and WASF3 [78,117,118]. The roles for Hsp90 in tumor cell autonomous metastatic properties, such as migration and invasion, are mediated through the chaperoning of a number of targets, most often tyrosine kinases, including Src and FAK [117,118,119,120].

#### 2.2.6. HSPs and Tumor Initiation

**Figure 4:**
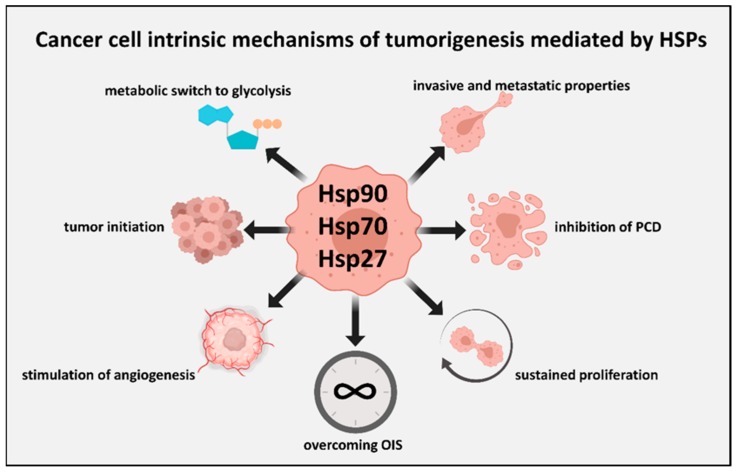
An overview of cancer cell-autonomous processes mediated by Hsp27, Hsp70, and Hsp90 that promote tumorigenesis. The transforming potential of cancer cell-autonomous events such as oncogenic mutations is commonly dependent on support from intracellular Hsp27, Hsp70, and Hsp90. The activities of key intracellular mitogenic signal transducers are also dependent on intracellular HSPs for sustained pathway activation, and thus HSPs mediate how cancer cells respond to the growth signals emanating from the tumor microenvironment. Similarly, signals from the tumor microenvironment can promote tumor-initiating properties within the carcinoma cells, the molecular features of which have also been closely linked with subpopulations enriched for Hsp72 and Hsf1. Such transforming events require the ability of tumor cells to overcome senescence (oncogene induced senescence—OIS), and the Hsp72 expression is required to overcome this hurdle to tumorigenesis. We also represent here other key tumorigenic processes supported by intracellular HSPs, including the inhibition of programmed cell death (PCD), altered cellular metabolism to favor glycolysis, stimulation of angiogenesis by cancer cells, and invasive and metastatic properties.

Tumor cell populations are characteristically heterogeneous, incorporating cancers derived from many tissues of cells of a different phenotype, as well as stem and progenitor cells [121]. Tumor initiation seems to be a property of only a fraction of the tumor cell population, with the majority of cells unable to seed tumors [122]. The remainder of the population may resemble differentiated cells and could play a support role in tumorigenesis, although evidence is mounting that such cells retain considerable plasticity and can be recruited into the tumor initiating fraction after treatments such as radiotherapy and chemotherapy, participating in resistance [123,124]. Tumor initiating cells (TIC) usually display some of the properties of tissue stem cells, express surface markers such as CD44 and Sca-1 characteristic of such cells, and appear to play key roles in tumor initiation and metastasis [121]. The heat shock system seems intimately involved in the phenotype of mammary TIC, which is highly enriched in activated HSF1 and its downstream products, such as Hsp72 and MTA1 [16,22]. HSF1 plays a key role in the TIC properties, in part by increasing the levels of nuclear beta-catenin, a key molecule in the maintenance of tissue stem cells [13]. The multiple oncogenic processes dependent on HSP overexpression are depicted in Figure 4.

## 3. HSPs and Tumor cell Extrinsic Properties of Tumors

### 3.1. Influence of the Extracellular Milieu and the Tumor Stroma on the Malignant Phenotype

Tumorigenesis and malignant progression may involve processes in addition to cancer cell intrinsic mechanisms, dependent on the sequential transforming mutations that overcome homeostatic regulation discussed above [69,125]. Increasing evidence suggests a key role for the tumor extracellular milieu, and infiltrating normal cells in further programming the tumor cells to grow and metastasize [126]. It was shown recently that Hsp70 ablation or the chemical inhibition of Hsp70 in host mice reduced tumorigenicity, and this was related to the reduced infiltration of macrophages into tumors [127]. The reduced tumorigenicity was related to the reduced mobility of the macrophages and the inhibition of the expression of MAP3K8, CXCR4, and FOXM1 [127]. There is also strong evidence to indicate the HSF1/heat shock system has an important role in modulating the transcriptional responses stimulated by the stromal–tumor interplay [128]. For example, HSF1 was shown to regulate transcription within tumor cells upon co-culture with fibroblasts, and within fibroblasts upon co-culture with tumor cells. Interestingly the cohort of HSF1-regulated genes was found to be distinct between the two cell types, yet each to have general pro-tumorigenic features. Specifically, Hsf1 bound the *Sdf1/Cxcl12* gene promoter and regulated its gene expression. The expression of *TGFB* genes was also found to be dependent on HSF1, although this seemed to be independent of the direct transcriptional regulation by HSF1 [128]. TGF-β and CXCL12 are key factors in the regulation of the tumor milieu, and can lead to the expression of extracellular matrix proteins such as fibrillar collagens known to be involved in invasion and metastasis [71]. The first indication of a role for HSPs in ECM assembly was in the study of Hsp47 encoded by the *SERPINH1* gene, an unusual HSP with a key role in collagen assembly and maturation [129,130]. Hsp47 has a versatile role in ECM assembly, and was shown to participate in the deployment of type I and II collagens, molecules that, when expressed to a high level, can lead to tumor desmoplasia and increased metastasis [108,126]. Indeed, Hsp47 has thus been shown to drive tumor growth and invasion through these interactions [131].

**Figure 5:**
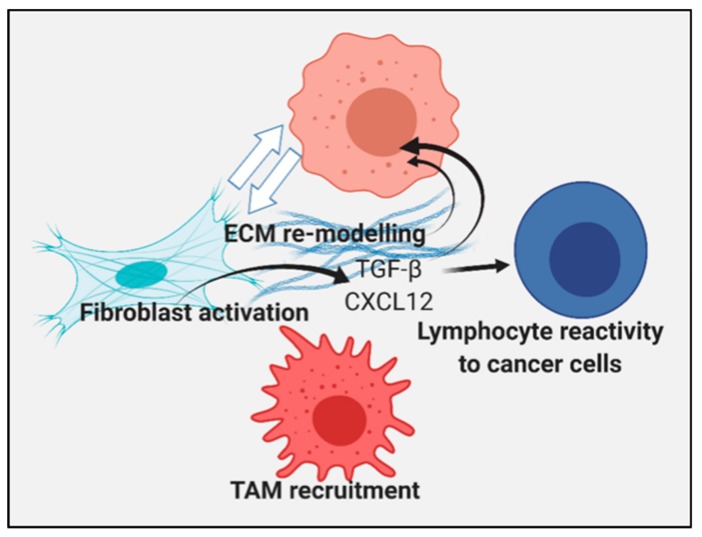
The activities of HSPs can promote a tumorigenic stroma. We represent cancer cells (at the top) within an extracellular stroma containing infiltrating cancer associated fibroblasts (CAFs) and tumor associated macrophages (TAMs) that affect the tumor microenvironment in an HSF1/HSP-dependent manner. The interplay between CAF and carcinoma cells is affected by the HSF1 expression in each of these cell types, and the HSF1 activity promotes the secretion of the TGF-β and CXCL12 factors, which lead to the secretion of ECM molecules, such as collagens, that can modulate tumor cell behavior by affecting the structure of the tumor milieu and binding to integrins on the cancer cell surface. TGF-β and CXCL12 can also influence the properties of the cancer cells and the reactivity of tumor infiltrating lymphocytes. Hsp72 has also been shown to be important for the recruitment of TAM, which can secrete growth promoting factors in an HSP-dependent manner.

The more conventional HSP family members also play roles in collagen synthesis and secretion in a range of cell types, including Hsp70 [132] and Hsp90 [133,134]. Hsp27 can be induced by TGF-beta, and is needed for type 1 collagen synthesis by myofibroblast, and plays a role in fibrosis [135]. Clearly, through these interactions, HSPs may play key roles in tumor growth and metastasis, beyond the conventional tumor intrinsic mechanisms (Figure 5).

### 3.2. Tumor Immunity

HSPs play powerful, although often ambiguous, roles in tumor immunity [136]. Because of their abilities to bind to a wide spectrum of peptide sequences, they have been used to prepare effective anti-cancer vaccines, in which tumor antigens are bound to HSPs, which can then direct them for processing by antigen processing cells, such as dendritic cells [137,138]. However, it is not clear whether tumor cell intrinsic HSPs are characteristically immunogenic or immunosuppressive. TGF-β and CXCL12 appear to modulate the relative populations of tumor infiltrating lymphocytes and their respective activities; this has been demonstrated by studies showing the blockade of TGF-β or CXCL12 to potentiate tumor responses to immune checkpoint blockades across multiple tumor models [71,139,140,141]. Thus, activation of the HSF1 > TGF-beta and CXCL12 mechanism might be predicted to suppress anti-tumor immunity, and may represent a beneficial feature of targeting HSF1 in cancer. In addition, it is known that HSPs can be released from tumor cells and can influence the behavior of the adjacent cells in this way [142]. Much of the Hsp70 or Hsp90 released by the tumor cells is contained in exosomal particles [143], and it has been shown that Hsp72- containing exosomes can lead to immunosuppression [144,145]. Most evidence therefore points to an immunoregulatory role for HSPs released from tumors and stromal cells under basal conditions. However, in the context of cytotoxic therapy, overexpressing Hsp70 in the treated tissue leads to profound, tumor specific immunity through the induced expression of IL-6 [146,147]. The effects of extracellular HSPs on immunity are therefore likely to be highly context dependent.

## 4. Extracellular HSPs

**Figure 6:**
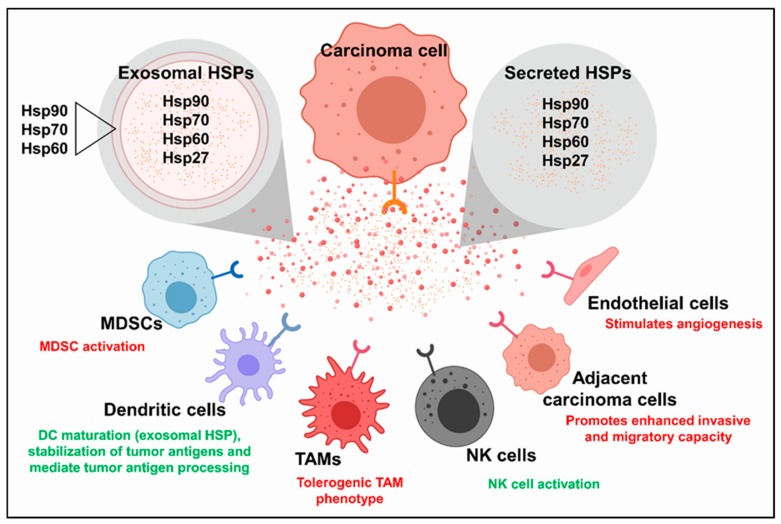
Tumor cells can modulate the tumor microenvironment by secreting HSPs. Carcinoma cells derived from several tissues have been identified to secrete HSPs into the extracellular space in soluble form or within exosomes. HSP species, including those listed above, are often abundant within the lumen of tumor cell-derived exosomes or within the exosomal membrane. HSPs secreted from carcinoma cells in soluble form or within exosomes can modulate the biology and functions of other cells in the tumor microenvironment. Specifically, secreted HSPs have been shown to modulate the activities of myeloid-derived suppressor cells (MDSCs), dendritic cells, TAMs, natural killer (NK) cells, other/neighboring carcinoma cells, and endothelial cells. Extracellular HSPs can promote tumorigenic processes (listed in red text), including immunosuppressive MDSC activity, carcinoma cell invasion and migration, and angiogenesis. Alternatively, extracellular HSPs can also promote tumor immunity (listed in green text) by stabilizing tumor antigens, stimulating TAMs to secrete inflammatory cytokines, and by mediating antigen processing by antigen presenting cells (APCs).

The established role of HSPs is, of course, as intracellular molecular chaperones, and we have listed an array of oncogenic molecules dependent on HSP overexpression, both in tumor and stromal cells. However, it is increasingly clear that HSPs do escape the cell in free form and in exosomes to interact with the receptors on self, on adjacent cells of the same lineage, or on different cell types (Figure 6). Similar to many intracellular HSPs, the extracellular production levels of several HSPs are increased by cancer cells of various origins, and in some cases, have been linked to greater degrees of disease burden [148,149,150,151,152]. A key further question in understanding the roles of HSPs in cancer then is the relative importance of intracellular and extracellular HSPs [153,154]. Each of the *HSP* gene families contains multiple paralogs, some of which could be specialized for an extracellular function. In the case of Hsp90, which exists as two different paralogs expressed in the cytosol, Hsp90-alpha and Hsp90-beta, the former seems specialized for extracellular secretion, while the latter is generally retained in cells and seems more important in the “housekeeping folding” of clients within the confines of the cell [154]. Secreted Hsp90 plays a powerful role in cancer cell invasiveness through both binding to the surface receptors such as LRP1/CD91, and interacting with matrix metalloprotease 2 on the cell surface to mediate invasiveness, EMT, and a TIC phenotype [155,156,157,158,159]. Interestingly, cell impermeant Hsp90 inhibitors can block these processes [160].

The HSP70 family is more extensive, and several HSP70 family members have been reported to be secreted by tumor cells [142,161]. The current evidence indicates that extracellular Hsp70 can either promote tumorigenic processes or have tumor-limiting activities, and can be targeted or utilized to potentiate tumor responses to treatment [148,162]. The tumor-limiting activities of the Hsp70 species described to date have primarily involved various methods of stimulating tumor immunity. For example, Hsp70-positive tumor derived exosomes promoted cytolytic natural killer (NK) cell activity towards tumor cells in a Hsp70-dependent manner [163]. Similarly, Xie et al. linked Hsp70-enriched exosomes to an increased NK cell activity, where they found exosomes isolated from J558 myeloma cells transgenically overexpressing Hsp70 stimulated dendritic cell (DC) maturation, and protection from J558 tumor cell challenge, an effect dependent on CD8 + T cell and NK cell activity [164]. Extracellular HSPs may also promote tumor immunity by facilitating DC functions through the stabilization of tumor antigens, and by enhancing tumor antigen processing by DCs. The tumorigenic properties of extracellular Hsp70 members have also involved immunomodulation. For example, exosomal Hsp72 was found to promote MDSC activation via binding toll like receptor-2 (TLR2), activating downstream IL-6 release, and subsequent STAT3 activation [144]. Hsp27 was shown to be present at high levels in the interstitial fluid of human breast tumors, and the differentiation of human monocytes to macrophages in the presence of soluble Hsp27 was demonstrated to induce tolerogenic and pro-angiogenic TAM properties in the cultured macrophages [165]. Hsp27 may also play powerful roles in programming the extracellular environment through triggering the release of IL1-beta, TNF-alpha, IL-10, PGE2, and VEGF-A [99,165,166]. Secreted Hsp27 may thus influence tumor immunity and angiogenesis, as well as the roles in metastasis mentioned above.

## 5. Interactions between Tumor Cells and HSPs in Treatment of Cancer: Targeting Molecular Chaperone Functions in Cell Survival and Tumor Cell Evolution

HSPs comprise a significant portion of the total (1%–10%) protein content in cancer cells [167]. The elevated levels of HSPs also maintain pro-growth signaling components, inhibit apoptosis, prevent replicative senescence, help create the immunosuppressive tumor milieu, rewire metabolic processes, and resist cytotoxic anti-cancer therapies [53,57,136,168,169,170,171,172] (Figure 4). Together, these properties provide cancer cells with distinct survival and proliferative advantages that incentivize continual malignant growth.

Most remarkable with regard to oncogenesis and the development of resistance, is the ability of chaperones to function as evolutionary capacitors, and enable the emergence of new traits [173,174]. Pioneering work in the laboratory of the late Susan Lindquist demonstrated that HSP90 stabilizes and maintains mutated quasi-stable client proteins that would typically be degraded [174,175]. Such protein variants bound by Hsp90 under normal conditions remain functionally dormant, but become active under proteotoxic stress conditions in which free HSP concentrations are limited, forcing Hsp90 to dissociate from these protein variants, and allowing them to display their altered function [174]. This concept may be most applicable to the study of germline variants within the human genome [176], where latent oncogenic variants would remain dormant, until exposure to stress strips away chaperone support and allows the variant to display its altered function and oncogenic potential.

Alternatively, Hsp90 also enables oncogenesis by supporting the increased enzymatic activity that can be caused by activating mutations. This concept is relevant to spontaneous mutations, and is demonstrated by the observation that protein kinases, when mutated to increase phosphorylation and resulting oncogenic activity, cause the dual lobe structure of kinase domains to become destabilized [177,178,179]. Hsp90 along with CDC37 stabilizes the structure of these mutated kinases, thereby enabling oncogenesis [43]. This support of “fast and loose” kinase activity helps drive oncogenesis. However, when the cells become stressed and HSP support is depleted, the mutated kinases misfold and lose activity—this is a part of the logic behind the use of most Hsp90 inhibitors [20].

The abilities of HSPs to support both germline and spontaneous genetic variation along with dramatic changes in gene expression define HSPs as oncogenic enablers. These properties also make HSPs facilitators of resistance to clinical cancer therapies. In tumors in which cancer cells are subjected to multiple rounds of cytotoxic regimens, the ability of HSPs to support myriads of clonal variants would allow for the eventual evolution of resistance to the therapy, a phenomenon commonly observed over the course of cancer treatments [180]. Understanding how to inhibit molecular chaperone function is therefore desirable in order to prevent the rise of resistant cancer cells by this mechanism. Moreover, in contrast to other cancer therapy targets, such as protein kinases and G-protein coupled receptors, whose genes number in the hundreds and have overlapping functions, there are only a handful HSP genes that service the entire proteome, making them keystones that hold together cellular protein networks.

Targeting HSPs has been an ongoing endeavor, although, their evolutionary significance effectively inhibiting HSPs without major toxicity to normal tissues has proven difficult [73,181]. The development of effective Hsp70 inhibitors has proven to be difficult, likely due to the unique orientation in which this molecule binds ATP and prevents access to small molecule inhibitors, coupled with the continuous occupation of the nucleotide-binding pocket. Upon ATP hydrolysis, co-chaperones allosterically exchange ADP for ATP [182] (Figure 2). This allosteric regulation of Hsp70 has, however, permitted the identification of inhibitors such as HS-72 [183]. Further leveraging the allosteric regulation of Hsp70, other small molecules have been discovered that function by disrupting key protein–protein interactions. For example, JG98 interferes with Hsp70–Bag3 interaction and Hsp70 ADP-exchange [184], while C86 prevents Hsp70–Hsp40 interaction and proper client loading on to Hsp70 [185]. Both drugs have shown potential for reducing tumor growth in prostate cancer xenograft models [185]. The direct targeting of the Hsp70 ATP-binding domain has also been successfully carried out, with the identification of VER155008 [186], which is capable of inhibiting Hsp70 activity, with enough potency to induce the aggregation of the nucleolus [187].

The discovery, almost 25 years ago, of the natural product Geldanamycin as a potent Hsp90 inhibitor that binds to the N-terminal ATP-cleft of the chaperone, hinted that multiple oncogenic signaling pathways could simultaneously be disrupted by this molecule [188]. However, the study of the relationship of Hsp90 with HSF1 has uncovered a drawback to these inhibitors, a feedback mechanism that amplifies the heat shock response. This results in the increased expression of Hsp90, along with numerous other HSPs, a reaction that can overcome the temporary inhibition, and the likely reason why Hsp90 N-terminal inhibitors have stalled in the phase III clinical trials [11]. Nevertheless, the subsequent use of Hsp90 N-terminal inhibitors, such as Ganetespib, may prove effective as agents for the treatment of neurodegenerative diseases caused by protein aggregation [189]. Another class of inhibitors that target the C-terminal domain of Hsp90 induces the degradation of client oncogenes, but does not activate the HSF1-feedback loop and continue to be developed [190], along with small molecules that specifically target the constitutively expressed Hsp90 Beta paralog [191,192]. Furthermore, as HSPs operate in relay and have redundant protein folding activities, another tactic to dramatically disrupt proteostasis is to simultaneously inhibit Hsp70 and Hsp90, an approach that has shown some merit [60,84].

Inhibiting the overall expression of HSP genes by targeting the master transcription factor HSF1 is, in concept, one of the best ways to combat cancer (Figure 1), as HSF1 also drives tumor malignancy through a cancer-specific transcription program that includes genes other than HSPs [23]. Directly targeting HSF1, however, is quite difficult because of its lack of a binding cleft and overall structure. As recently reviewed by Kijima and colleagues [193], the agents that target the up- and down-stream processes may be a potential way to indirectly inhibit HSF1. Kinases that help activate HSF1, such as mTOR and MEK1, can be inhibited by clinically approved drugs, while the helicase/translocase, XPB, which helps initiate stress induced transcription, is blocked by the triptolide analogs that are currently entering phase II trials for the treatment of pancreatic cancer [194].

Another option for disrupting proteostasis and overwhelming the chaperome is proteasome inhibition. Consequently, proteasome inhibitors have been widely investigated, leading to the clinical application of bortezomib for the treatment of multiple myeloma and mantle cell lymphoma [195,196]. Degrading misfolded proteins instead of allowing HSPs to refold them, provides cancer cells an alternative channel to maintain proteostasis and the bypass inhibition of HSPs. Indeed, combining proteasome and Hsp90 inhibitors leads to enhanced therapeutic responses, indicating the suppression of a resistance pathway [197,198].

## 6. Conclusions

(1) The heat shock response appears to be switched on in a wide variety of cancer cells, although the mechanisms involved in triggering HSP induction in tumors remains unclear. The activated HSF1 and increased levels of HSPs observed in tumors are each associated with a poor prognosis for patients. In certain cancers, the major HSPs and co-chaperones appear to bind tightly into large multiprotein chaperoning complexes, which comprise the epichaperome and may be essential in enhancing the malignant phenotype. The current studies indicate a systemic change in chaperome function in transformed contexts. Thus, both altered HSP expression levels and a greater functional and physical integration between individual chaperome entities are a feature of many human tumor cells.

(2) Members of the small HSP family, the Hsp70 family, and Hsp90 proteins each play key individual roles in mediating the cancer cell intrinsic traits that contribute to tumorigenesis, such as unscheduled cell division, escape from programmed cell death and senescence, de novo angiogenesis, and increased invasion and metastasis. Increased HSP expression thus seems a powerful governor of a cancer cell’s autonomous capacity to promote tumorigenesis.

(3) There also appears to be key roles for HSF1 and its products in cancer cell extrinsic mechanisms of tumorigenesis, and HSPs may permit essential pathways of pro-malignant cell programming by cells in the tumor milieu, to provide a fostering environment for tumor growth.

(4) Through a reciprocal mechanism, tumor cells can also program their own environments by the release of HSPs into the extracellular spaces. Such HSPs can then bind to the receptors on adjacent cells and promote EMT and metastasis in tumor cells, as well as triggering effects on the cells of the immune system.

(5) Increased levels of extracellular HSPs may participate in establishing the immunosuppressive tumor milieu, and thus further support the establishment of tumors and metastases.

(6) As stress proteins, HSPs protect tumor cells from cytotoxic therapy, and as molecular chaperones, they can also permit the evolution of resistant traits by permitting the accumulation of quasi-stable variant proteins. These disparate mechanisms combine to make HSPs important factors in transformation and tumor progression, and key targets for enhancing cancer therapy.

(7) Controlling the expression and activity of HSPs is critical to preventing their enabling of oncogenesis and further malignant growth. However, targeting individual HSPs with small molecules has not proven to be highly effective in the clinic because of feedback loops within the heat shock response and alternative mechanisms to maintain proteostasis. Combining multiple proteostasis disruptors with other targeted therapies might be indicated.
